# Anatomical Study of the Communication Between the Mental Nerve and Marginal Mandibular Branch of the Facial Nerve

**DOI:** 10.7759/cureus.33733

**Published:** 2023-01-13

**Authors:** Joe Iwanaga, Naotaka Kishimoto, Soichiro Ibaragi, Juan J Cardona, Arada Chaiyamoon, Mi-Sun Hur, Yoshiaki Nakamura, Jingo Kusukawa, R. Shane Tubbs

**Affiliations:** 1 Department of Neurosurgery, Tulane University School of Medicine, New Orleans, USA; 2 Department of Neurology, Tulane University School of Medicine, New Orleans, USA; 3 Department of Oral and Maxillofacial Anatomy, Graduate School of Medical and Dental Sciences, Tokyo Medical and Dental University, Tokyo, JPN; 4 Dental Anesthesiology, Faculty of Dentistry and Graduate School of Medical and Dental Sciences, Niigata University, Niigata, JPN; 5 Department of Oral and Maxillofacial Surgery, Graduate School of Medicine, Dentistry and Pharmaceutical Sciences, Okayama University, Okayama, JPN; 6 Department of Anatomy, Khon Kaen University, Khon Kaen, THA; 7 Department of Anatomy, Catholic University of Daegu School of Medicine, Daegu, KOR; 8 Department of Dental and Oral Surgery, Saiseikai Hita Hospital, Hita, JPN; 9 Department of Dental and Oral Surgery, Dental and Oral Medical Center, Kurume University School of Medicine, Kurume, JPN; 10 Department of Structural and Cellular Biology, Tulane University School of Medicine, New Orleans, USA; 11 Department of Surgery, Tulane University School of Medicine, New Orleans, USA; 12 Department of Anatomical Sciences, St. George’s University School of Medicine, St. George’s, GRD; 13 Department of Neurosurgery, Ochsner Neuroscience Institute, Ochsner Health System, New Orleans, USA

**Keywords:** cadaver, anatomy, communication, facial nerve, mental nerve

## Abstract

Background

Trigeminal-facial nerve communications have been recognized for over 100 years. More specifically, the mental nerve (MN) and marginal mandibular branch of the facial nerve (MMb) communication have been studied but the termination of these branches remains unclear. Therefore, we aim to classify the anatomical communication between the MN and MMb by its course.

Methods

Sixty sides from thirty adult cadaveric heads were dissected. The communicating branches were dissected and observed anatomically and histologically.

Results

Communication between the MN and MMb was found on all sides. Based on the course, the communication was classified into two types, superior and anterior. For the superior type, a small branch of the MN and MMb join and travel superiorly to reach the lower lip area. The communication was observed at the level of the mental foramen or above it. For the anterior type, a small branch of the MN and MMb join and travel anteriorly to reach the chin area. The termination was either in the subcutaneous tissue of the chin or in the mentalis. This communication was observed below the mental foramen. Histological observation revealed that these communications contained two or more perineuria.

Conclusions

Although the function of such neural communications is still unclear, this study helps better understand the anatomical variants of these unions and provides a novel classification system.

## Introduction

Trigeminal-facial nerve communications have been recognized for over 100 years. For example, Dixon [1] commented on the potential importance of such connections between cranial nerve (CN) V, VII, and IX in 1899. Martin and Helsper [2] stated that although the significance of the communications between CN V and VII have not been functionally established, such communications would not be present without a purpose. In 1974, Baumel [3] focused on the function of such intercommunications in the innervation and reinnervation of the muscles of facial expression. Communication between other branches of CN V such as the buccal nerve and CN VII has also been described in the literature [4]. More specifically, Rödel and Lang [5] observed the connection between the mental nerve (MN) and the marginal mandibular branch of the facial nerve (MMb), and Hwang et al. [6] conducted an anatomical and histological study of this communicating branch. There are various hypotheses regarding the function of these communications. For example, some suggested that facial nerve fibers might use alternative pathways such as along trigeminal nerve branches to reach its target muscles [3].

Since previous studies have only demonstrated such trigeminal/facial nerve communications [6], our study aims to classify these anatomical communications between the MN and MMb.

## Materials and methods

Sixty sides from 30 adult Caucasian cadaveric heads (12 females and 18 males) were used in this study. All 30 heads were formalin-fixed. The age of the specimens at death ranged from 57 to 98 years with a mean of 77.3 years. Specimens showing obvious surgical scars were excluded from the study.

Communications between the MN and MMb of the facial nerve were identified and dissected. All dissections and measurements were carried out by two anatomists (Joe Iwanaga and R. Shane Tubbs) using a surgical microscope (OPMI CS NC31, Carl Zeiss, Oberkochen, Germany). All measurements were made using a digital microcaliper (Mitutoyo, Japan). Communications (nerve fibers immediately after merging) were excised from three randomly selected sides and histological analysis (Masson-trichrome stain) was performed. The slides were then observed with a light microscope and images were captured with a digital imaging capture device. Statistical analysis between sides and sexes was conducted using a t-test with significance set at p<0.05.

The present study protocol did not require approval by the ethics committee of our institutions and was performed in accordance with the requirements of the Declaration of Helsinki [7].

## Results

**Figure 1:**
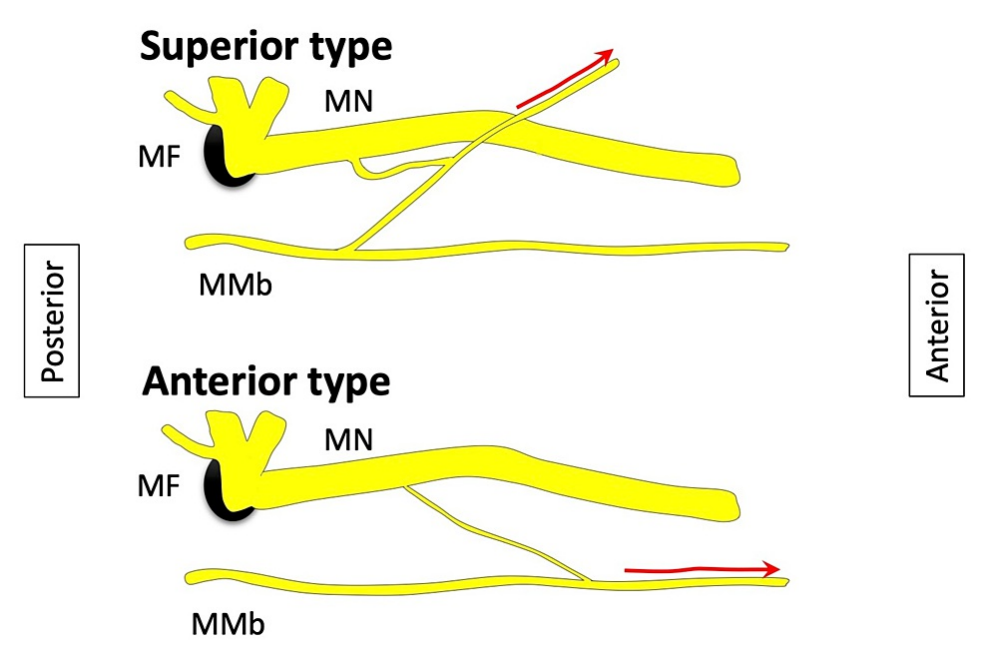
Schematic drawing of the two types of communication. MN: mental nerve; MMb: marginal mandibular branch of the facial nerve; MF: mental foramen

**Figure 2:**
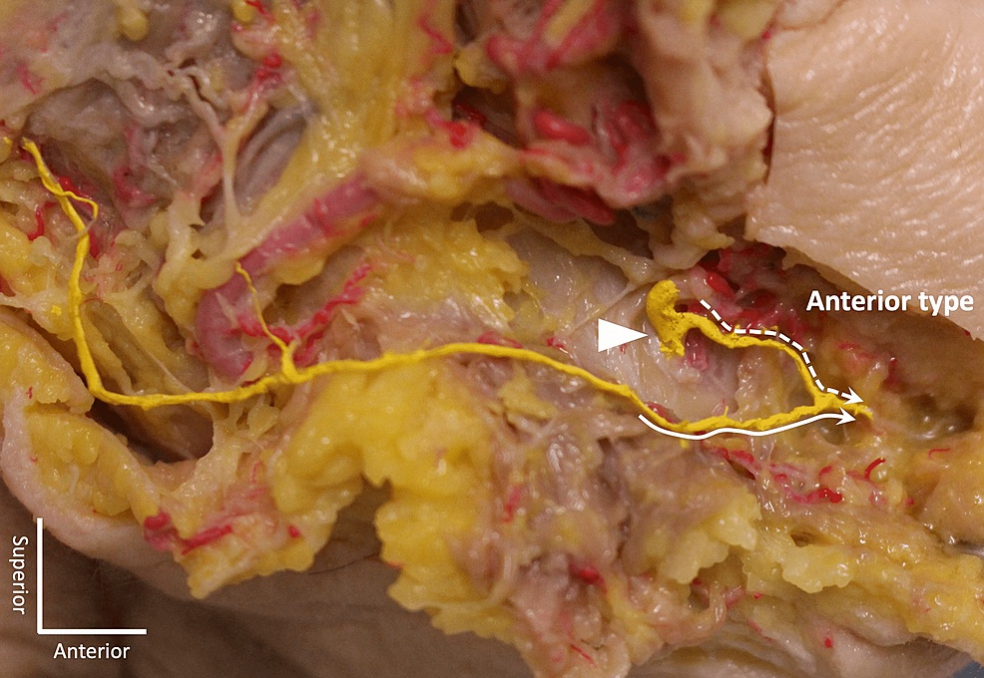
An example of one communication on the right side. Arrow: mental foramen; Dotted line: mental nerve; Continuous line: marginal mandibular branch of the facial nerve

**Figure 3:**
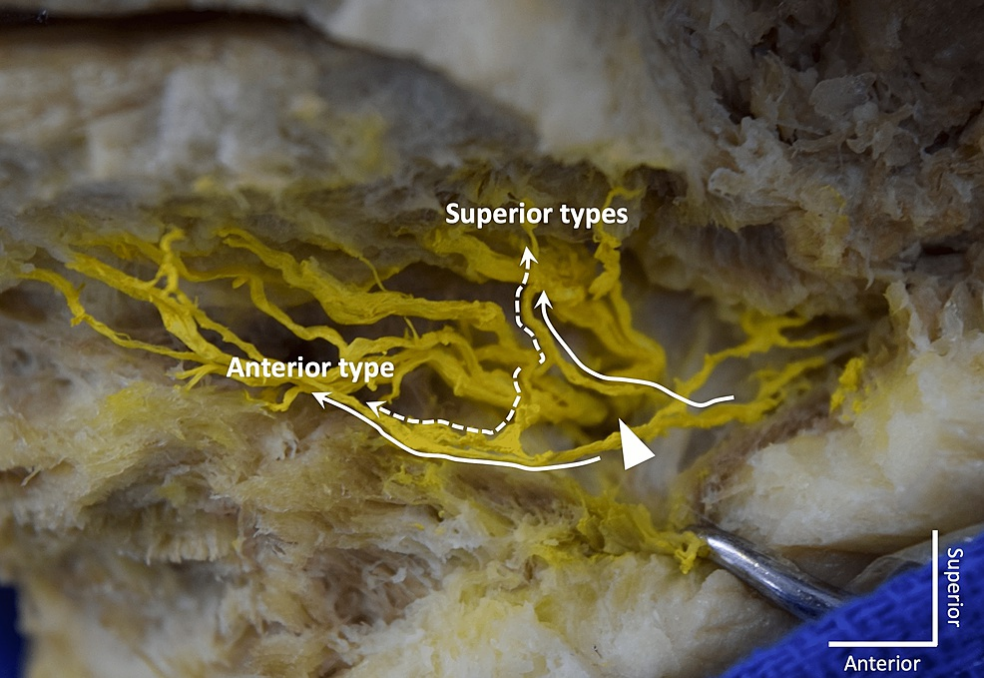
An example of two communications on the left side. Arrow: mental foramen; Dotted line: mental nerve; Continuous line: marginal mandibular branch of the facial nerve

**Figure 4:**
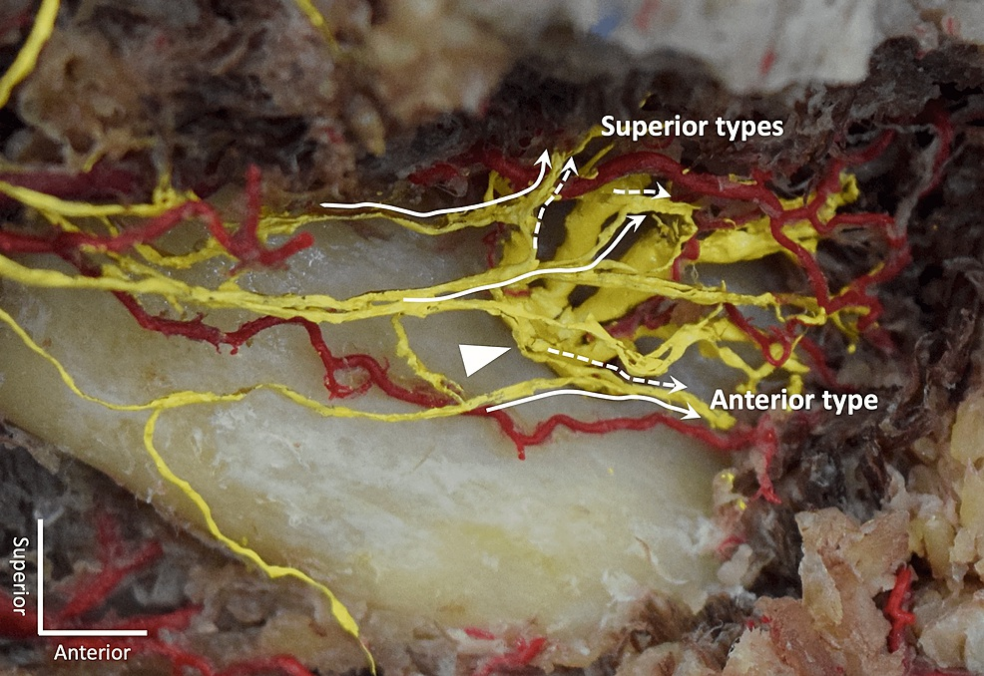
An example of three communications on the right side. Arrow: mental foramen; Dotted line: mental nerve; Continuous line: marginal mandibular branch of the facial nerve

The number of MMb was 1 to 3 on each side. Communications between the MMb and MN were found on all sides (100%, 60/60). Communication pattern was classified into two groups as seen in Figures 1-4.

Superior type

Small branch of the MN and MMb join and travel superiorly to reach the lower lip area. The communication was observed at the level of the mental foramen or superior to it.

Anterior type

Small branch of the MN and MMb join and travel anteriorly to reach the chin area. The termination was either in the subcutaneous tissue of the chin or to the mentalis. The communication was observed inferior to the mental foramen.

**Figure 5:**
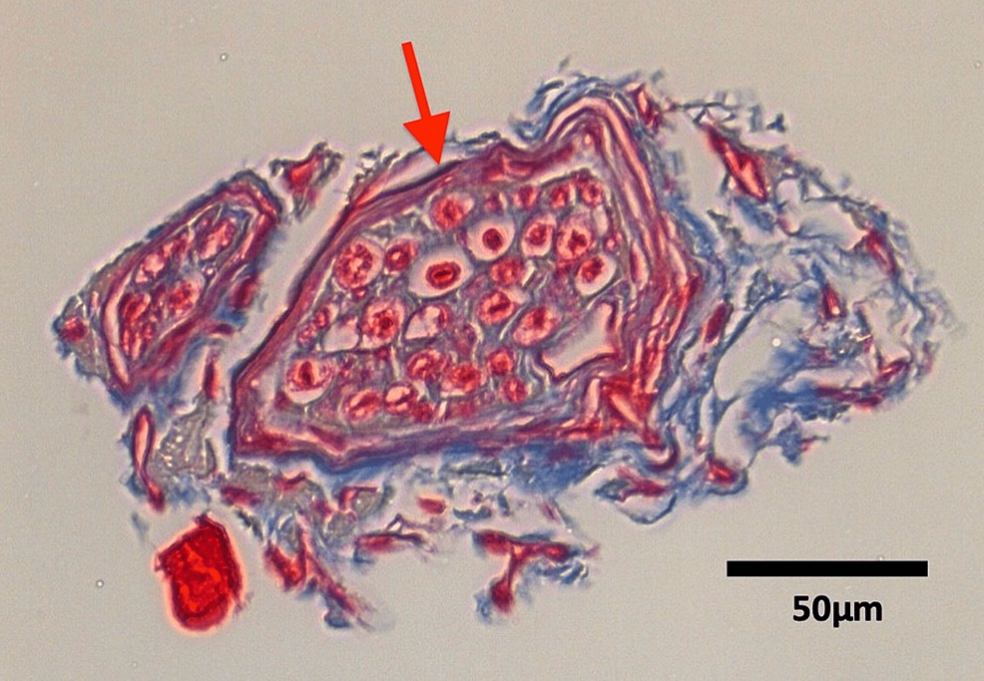
Masson-trichrome stain of the communicating branch of the superior type. Arrow: epineurium.

**Figure 6:**
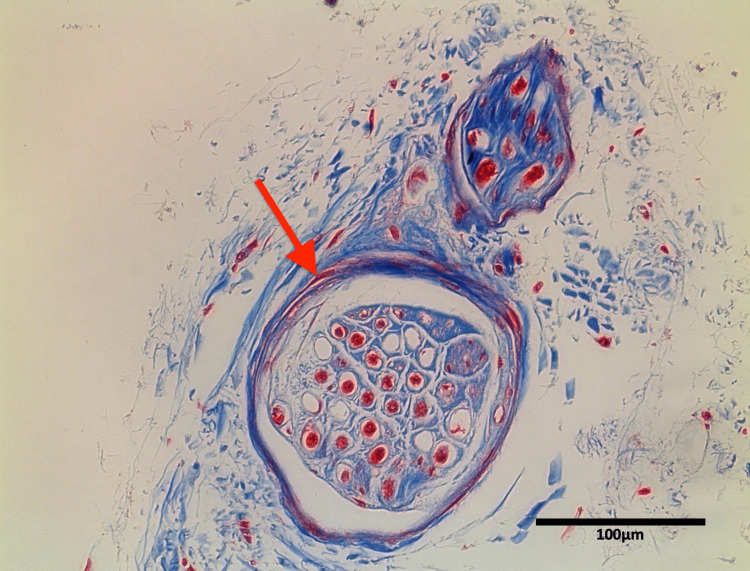
Masson-trichrome stain of the communicating branch of the anterior type. Arrow: epineurium.

The number of communications on each side ranged from 1 to 6 with a mean of 2.6. The anterior type communication was observed in 100% (60/60 sides, 1 to 2 per side). The superior type was observed in 70% (42/60 sides, 0 to 4 per side). The diameter of the communicating branch at the confluence ranged from 0.1 to 1.0 mm with a mean of 0.4 mm. Masson-trichrome stain showed that the two or more perineuria were observed within one epineurium (Figures 5-6). There was no significant difference in diameter or number of communicating branches between sex or side (p>0.05).

## Discussion

We identified connections between the MMb and MN and classified these into two types. Other similar connections have been reported. For example, the superior labial branch of the infraorbital nerve (CN V2) and facial nerve has been shown to join and form a nerve plexus [8]. Additionally, the supraorbital nerve (CN V1) and temporal branch of the facial nerve are reported to have interneural communication in 44% of cases [9]. Kwak et al. [10] reported communications between the auriculotemporal nerve (CN V3) and facial nerve in 93.3% of specimens. Martin and Helsper [2] reported that the communications between the branches of CN V and VII were closely associated with their motor endplates. It is believed that proprioception is transmitted via the cutaneous nerve innervating the skin over the mimetic muscles so that these fibers from the mimetic muscles probably travel centrally to the trigeminal nuclei in the brainstem [10,11].

In the present study, two different types of communication, i.e., superior type and anterior type, were identified. The superior type ended in the lower lip and the anterior type in the chin area.

Superior type (branch of the MMb joining MN)

If the MMb contains sensory fibers in its communicating branch, the superior type probably innervates the lower lip via the inferior labial branch of the MN [12-14]. If the MMb only contains motor fibers in its communicating branch, it probably travels within the common sheath with the MN branch and might function as the motor branch of the mimetic muscle of the lower lip covered by the skin which is innervated by the MN branch.

Anterior type (branch of the MN joining MMb)

If the MN contains motor fibers in its communicating branch, the anterior type probably innervates the chin area as the MMb does [15]. If the MN only contains sensory fibers in its communicating branch, it probably travels within the common sheath with the MMb and might function in proprioception of mimetic muscles of the lower lip along with adjacent motor fibers. 

Clinical significance

As Martin and Helsper [2] stated, communications could hardly be present without a purpose or function. Although it is challenging to avoid injury to the communication due to its relatively small diameter (mean 0.4 mm) and its number (1 to 8 with a mean of 2.6), surgeons should be aware of this anatomy during regional surgical/invasive procedures to this region. Injury to such a communicating branch might result in the same outcome as direct injury to the MN and/or MMb.

In clinical dentistry, the infiltration of anesthesia into the buccal gingiva of the premolar area often results in temporary paralysis of the lower lip (orbicularis oris). In addition to the direct infiltration of the anesthetics into the MMb, neural intercommunications might carry the anesthetics from the MN to the MMb which might cause paralysis of the lower lip. This hypothesis paves the way for further research into the incidence of lower lip paralysis in post-op patients after MN block or mandibular block.

Limitations

There are limitations to this study. The main parts of the incisive labii inferioris, depressor labii inferioris, and depressor anguli oris muscles were destroyed in order to observe nerve branches. Therefore, the exact innervation to these muscles, if present, was not observed. Lastly, the accessory mental foramina (AMF) were not focused on during this dissection. Some AMFs might have been missed as they might appear in around 10% of specimens [16,17].

## Conclusions

Sixty sides from cadaveric specimens were dissected and communication between the MN and MMb was observed on all sides. This study helps better understand the anatomical variants of these unions and provides a novel classification system categorizing the communication into two types, superior (70%) and anterior (100%). The former reaches the lower lip area and the latter, the chin area. Due to these neural intercommunications, the MN or MMb can be affected in cases where communicating branches are injured. In addition, these junctions are pathways for anesthetics to be directed to the MMb through the MN, causing either temporary or permanent paralysis of the lower lip.
